# Supplementary material for: How frictional slip evolves
Source: Nat Commun. 2023 Dec 14;14:8291. doi: 10.1038/s41467-023-44086-1 (PMC10719317; doi:10.1038/s41467-023-44086-1)
Supplement: Supplementary file 1 — Supplementary information [file 41467_2023_44086_MOESM1_ESM.pdf]

Supplementary Information for “How frictional slip evolves”

Songlin Shi[1], Meng Wang[1], Yonatan Poles[1], Jay Fineberg[1]*

[1]The Racah Institute of Physics, The Hebrew University of Jerusalem,

Givat Ram, Jerusalem 91904, Israel

Correspondence to: jay@mail.huji.ac.il

This file includes: Supplementary Figs. 1 to 6, Supplementary Table 1.

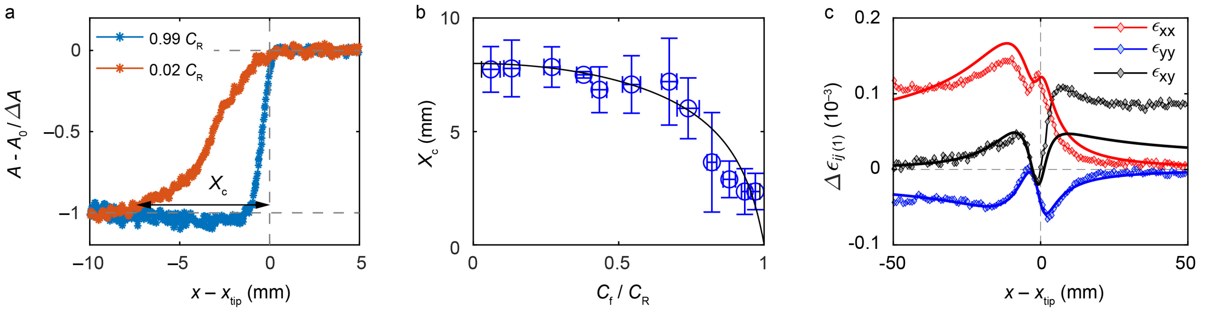

Supplementary Fig. 1. Characterization of the cohesive zone size. a, As $A(x,t)$ transitions from unbroken to broken contacts, the normalized contact area $(A - A_0)/\Delta A$ changes in space. The spatial scale of this transition, X_c, is a measure of the cohesive zone size. We define X_c as the spatial scale at which 98% of the normalized contact area drops. X_c varies with C_f. Examples for $C_f = 0.02C_R$ (red) and $C_f = 0.99C_R$ (blue) are shown. **b**, The relation between the cohesive zone size X_c and C_f/C_R. Measured values (squares) are compared to the theoretical contraction (black line), where the only input is $X_c(C_f = 0) =$ 8 mm. The error bars represent the standard deviations of X_c and C_f/C_R, respectively. **c**, Comparison of the functional form of elastic strain measured (strain gauges, dots) immediately above the grooved location at $X = 100$ mm, and the strains predicted by the LEFM using the cohesive zone model described in Methods (solid lines). The fracture energy used in the comparison is $\Gamma = 1.3$ J m^{-2}, which was obtained from the fitting the slip function in Fig. 2e. The red, blue and black colors represent the *xx*, *yy* and *xy* components of the strain, respectively.

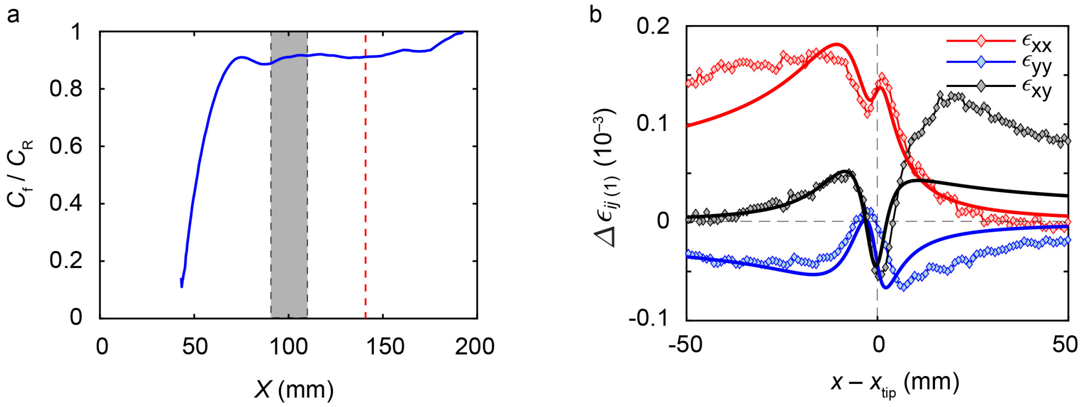

Supplementary Fig. 2. Evaluation of the influence of interface grooves. a, An example of the front velocity $C_f(X)$ of the first rupture along an interface (corresponding to the experiment shown in Fig. 1c in the main text). The gray shadow indicates the grooved region. The front velocity increased rapidly at the initial stage and then stabilized when approaching $0.9C_R$. It is clear that the grooves do not noticeably perturb the propagation process. **b**, The measured strain components at $X = 140$ mm (area without grooves) and the LEFM-predicted form (when coupled to the cohesive zone model described in Methods). We note that this fit was obtained with the same value $\Gamma = 1.3$ J m^{-2} as used at $X = 100$ mm (located above the grooved section), as shown in Supplementary Fig. 1**c**. The differences between the strains here and those in Supplementary Fig. 1c result from the slightly different values of C_f at the $X = 100$ mm and the $X = 140$ mm locations.

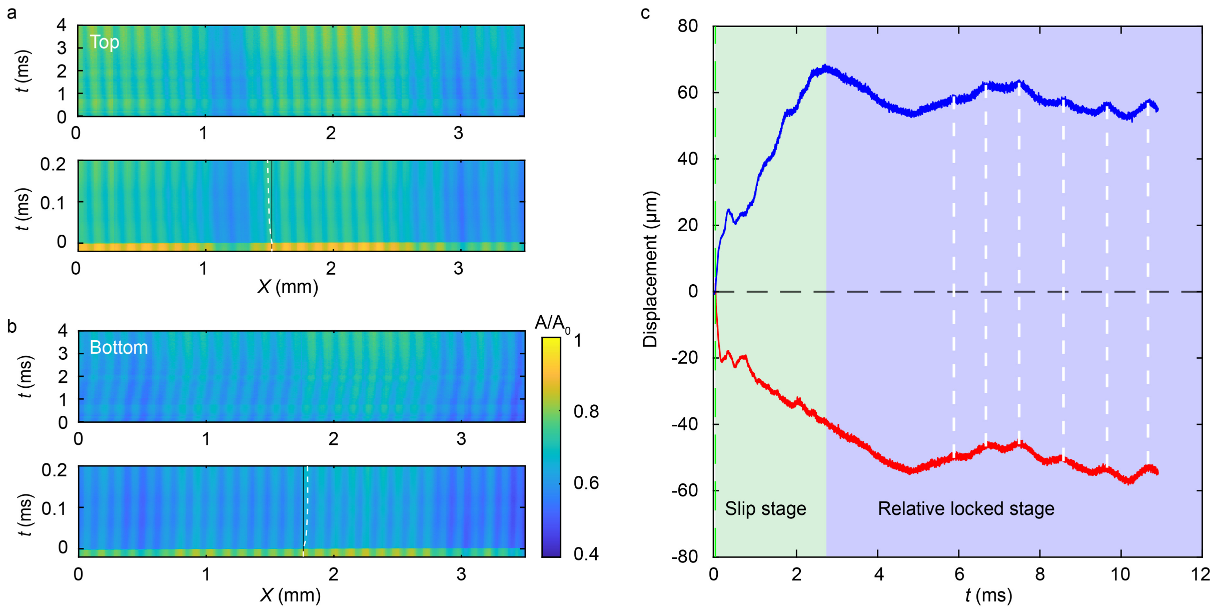

Supplementary Fig. 3. Details of groove patterns and long-time displacement. The motion of the groove patterns with time **a** (upper block) and **b** (lower block) over 4 ms (upper panels) and 0.2 ms (lower panels). $A(x, z, t)$drops significantly upon the first rupture's passage (t = 0 ms). The color bar represents the normalized contact area drop of both **a** and **b**. **c**, Variation of u^+ and u^- with time. Slip takes place over the first 3 ms, when displacements of two blocks are roughly antisymmetric. After 3ms, although the displacements of both blocks continue to vary, the slip, $u = u^+ - u^-$ is constant as the two blocks are locked to one another. During this locked stage, an approximate periodic oscillation with a 0.95 ms period persists (peaks indicated by white dashed lines). This motion is the result of a mechanical resonance of the loading system.

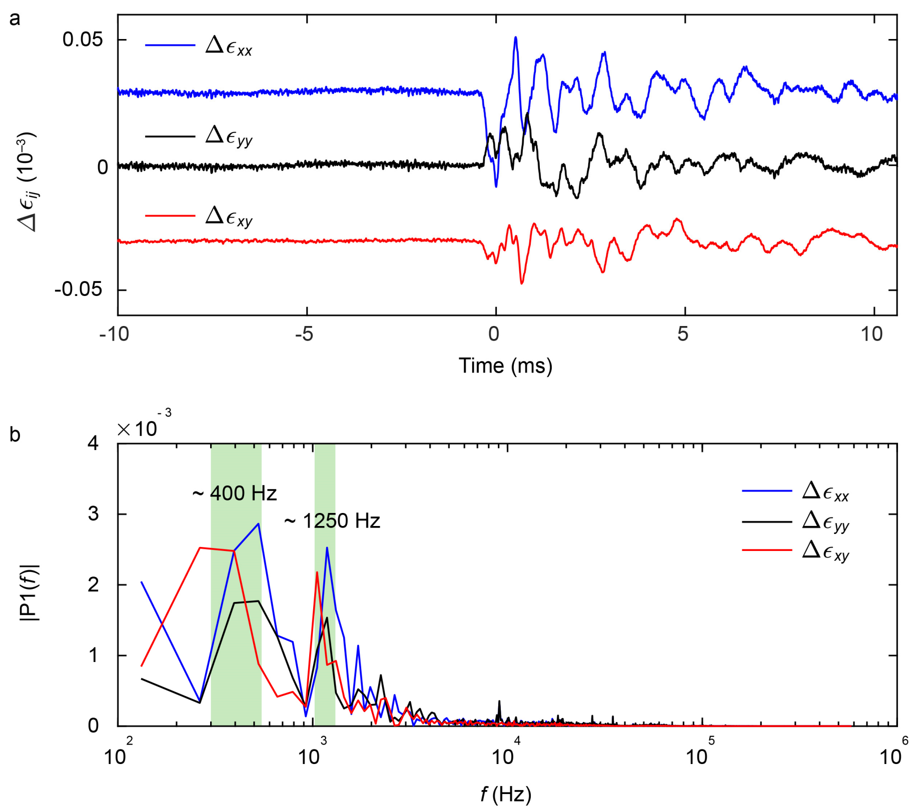

Supplementary Fig. 4. Resonant timescale of the mechanical system. a, The response of strain located at $x = 100$ mm after mechanically striking the system at applied values of F_N and F_S that were slightly below the onset of stick-slip motion. The three ε_{ij} components are shifted in y to enable comparison. **b**, The power spectrum P1 of the three time-series presented in **a**. The spectrum reveals that the system's resonant frequencies are approximately 0.8 ms (1250 Hz) and 2.5 ms (400 Hz). The red, blue and black lines represent ε_{xx}, ε_{yy}, and ε_{xy}, respectively.

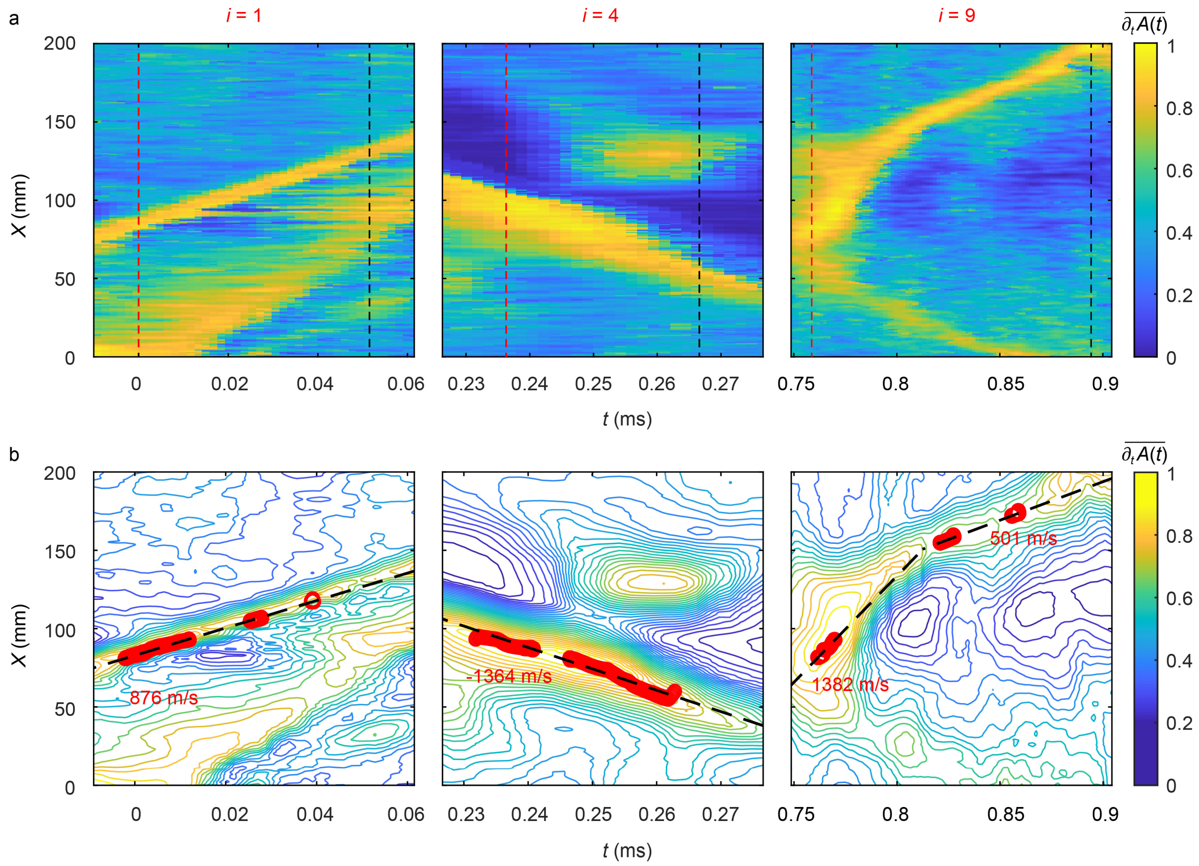

Supplementary Fig. 5. Determining the speed of (weak) secondary ruptures. a, Close up views of the normalized $\overline{\partial_t A(x,t)}$ measurements for the 1st, 4th, and 9th ruptures that were highlighted in Fig. 2a. These differentiated contact area maps accentuate the changes made by weak secondary rupture fronts, enabling both detection and measurement of their velocities. **b**, Contour maps of the images in **a.** Ridges of the contours (red dots denote clear points along a ridge) clearly delineate the rupture fronts. Rupture speeds (slope of the ridges) are obtained by the localized linear fitting (black dashed line) of the x, t paths of the ridges. The values of C_f obtained by this method are labeled. Note that in the panels on the right, corresponding to $i = 9$, the rupture is initially a supershear rupture ($C_\mathrm{f} = 1.3C_\mathrm{S}$) that transitioned to a sub-Rayleigh rupture ($C_\mathrm{f} = 0.47C_\mathrm{S}$).

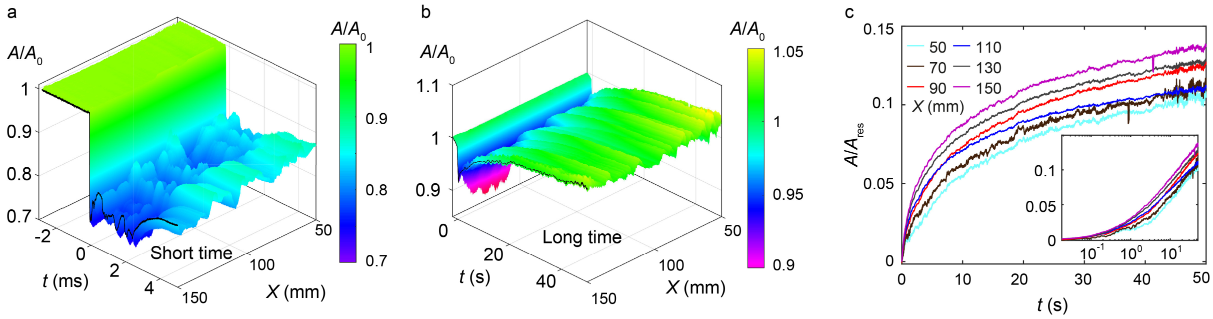

Supplementary Fig. 6. Contact area changes over time. a. 3D map of the contact area changes over the first 4.5 ms. $A(x, t)$ is normalized prior to the first (primary) rupture. **b**. 3D map of the contact area changes in the 50 seconds following rupture propagation. The ruptures create a non-uniform contact area drop and then heal via contact aging. The healing does not 'homogenize' the contact area changes along the interface. **c**. Logarithmic aging of the contact area after the cessation of slip (A/A_{res}, where A_{res} is the residual contact area immediately upon the passage of all secondary ruptures) at various spatial positions ($X \in [50, 150]$ mm) from **b**. The inset image is plotted in logarithmic time. The long-time aging rates are identical at each location.

Supplementary Table 1. The order, speed, contact area drop and fracture energy of sequenced ruptures.

Order of the ruptures	Rupture speed (m/s)	Contact area drop	Fracture energy (J/m^2)
1	876	0.2421	1.300
2	1547	0.0093	0.050
3	1206	0.0090	0.048
4	-1364	0.0438	0.235
5	-1335	0.0106	0.057
6	-1860	0.0014	0.008
7	--	0.0018	0.010
8	--	0.0039	0.021
9	1382	0.0346	0.186
10	-1430	0.0117	0.063
11	-959	0.0074	0.040
12	-1208	0.0020	0.011
13	-856	0.0054	0.029
14	--	0.0011	0.006
15	--	0.0029	0.016
16	--	0.0149	0.080
17	824	0.0194	0.104
18	-1252	0.0019	0.010
18	-899	0.0055	0.029
20	--	0.0037	0.020
21	--	0.0045	0.024
